# Determinants of Posterior Corneal Biometric Measurements in a Multi-Ethnic Asian Population

**DOI:** 10.1371/journal.pone.0101483

**Published:** 2014-07-09

**Authors:** Marcus Ang, Wesley Chong, Huiqi Huang, Tien Yin Wong, Ming-Guang He, Tin Aung, Jodhbir S. Mehta

**Affiliations:** 1 Singapore National Eye Centre, Singapore, Singapore; 2 Singapore Eye Research Institute, Singapore, Singapore; 3 Department of Ophthalmology, National University of Singapore, Singapore, Singapore; 4 Office of Clinical Sciences, Duke- National University of Singapore Graduate Medical School, Singapore, Singapore; 5 State Key Laboratory of Ophthalmology, Zhongshan Ophthalmic Center, Guangzhou, China; University of Missouri-Columbia, United States of America

## Abstract

**Purpose:**

To describe the corneal and anterior segment determinants of posterior corneal arc length (PCAL) and posterior corneal curvature (PCC).

**Methods:**

Cross-sectional, population-based study of 1069 subjects (1069 eyes) aged 40–80 years, from three major Asian ethnic groups. All underwent anterior segment optical coherence tomography imaging and analysis with Zhongshan Angle Assessment Program. Our main outcome measures were determinants of PCAL and PCC using adjusted, multivariate linear regression analysis, adjusted for confounders to obtain the estimated marginal means (EMM) with standard error (SE).

**Results:**

The overall mean (± SD) of PCC was: 6.51±0.39 mm; and PCAL was: 12.52±0.59 mm. Malays had a relatively longer PCAL (EMM = 12.74 mm, SE = 0.04 mm) than Chinese (EMM = 12.48 mm, SE = 0.03 mm, P<0.001), and Indians (EMM = 12.42 mm, SE = 0.03 mm, P<0.001). Anterior segment parameters had weak-moderate correlations with PCAL, which included: anterior chamber depth (ACD) (r = 0.55, P<0.001), PCC (r = 0.27, P<0.001), anterior corneal curvature (ACC) (r = 0.14, P<0.001) and central corneal thickness (CCT) (r = −0.07, P = 0.023). In multivariate analysis, anterior segment parameters explained only 37.6% of the variance of PCAL, with ACD being the most important determinant (partial R^2^  = 0.300; P<0.001). The determinants of PCC included ACC, PCAL and CCT (explaining 72.1% variation of PCC), with ACC being the most important determinant (partial R^2^  = 0.683; P<0.001).

**Conclusion:**

There was moderate correlation of PCAL with ACD, but anterior segment parameters accounted for only a small proportion of the variation in PCAL. The significant differences in PCAL and PCC amongst different Asian ethnic groups suggests that there is a need to consider this factor when planning for anterior segment surgeries such as endothelial keratoplasty.

## Introduction

Accurate measurement of the posterior corneal surface is important in the planning and execution of endothelial keratoplasty, which is increasingly becoming the surgery of choice in endothelial disease.[1] One way to more precisely image the cornea is with the use of anterior segment optical coherence tomography (AS-OCT, Visante; Carl Zeiss Meditec, Dublin, CA).[2], [3] However, measurements of anterior segment parameters from AS-OCT images are often inaccurate due to subjective placement of calipers or measurement tools.[4] The Zhongshan Angle Assessment Program (ZAAP, Guangzhou, China) has been shown to reliably derive these parameters from AS-OCT images,[5], [6], [7], [8] with high inter- and intra-observer agreement.[5], [6] The ZAAP software works by extracting the gray-scale images from the AS-OCT images and uses image processing and algorithms to define the anatomical landmarks.[5] This enables users to obtain rapid and objective analysis of anterior segment images for surgical planning and diagnosis of various ocular diseases (ZAAP research software available upon request; none of the authors have any commercial interest).[2], [3], [9] The ZAAP analysis obtains standard parameters such as central corneal thickness (CCT), anterior chamber depth (ACD), anterior corneal curvatures (ACC) using image-processing algorithms. [5]

Parameters derived from ZAAP include the posterior cornea curvature (PCC) and posterior corneal arc length (PCAL).[6] PCC is defined as the radius of curvature of the posterior surface of the cornea,[6] while PCAL is defined as the arc distance of the posterior corneal border between scleral spurs,[7] both reliably derived by ZAAP analysis from AS-OCT images in both normal and corneas with bullous keratopathy. [7] The clinical application of PCAL has been demonstrated in optimum donor graft size selection for endothelial keratoplasties.[7] The PCC and power of the posterior cornea surface is also important in planning for refractive procedures, phakic intraocular lens (IOL) implantations,[10] refractive changes after endothelial keratoplasty,[11] and determining the effect of the posterior surface on the total corneal astigmatism and higher-order aberrations (HOAs).[12] Studies have also shown that measuring the posterior corneal power and total corneal power may be important for IOL calculations, especially after corneal refractive surgery.[13], [14]

There are currently few studies that specifically describe posterior corneal parameters and their determinants. Therefore in this study, we analyzed the determinants of posterior cornea parameters such as PCAL and PCC, and their relationship with other anterior segment parameters using data from a large population-based study. We also describe differences in PCAL and PCC amongst three major Asian ethnic groups (Chinese, Malay and Indian).[15]

## Methods

### Study population

This population-based study involved adults aged 40–80 years under the Singapore Epidemiology of Eye Disease Study: the Singapore Malay Eye Study (SiMES, 2004–2006), the Singapore Indian Eye Study (SINDI, 2007–2009), and the Singapore Chinese Eye Study (SCES, 2009–2011). The details of each study methodology have been described.[15], [16] In brief, we used an age-stratified random sampling to select ethnic Malays, Indians, and Chinese living in Singapore during each stipulated study period. For this AS-OCT study, subjects who met the study eligibility criteria were systematically sampled (every fifth subject) as already described a priori.[15] We excluded subjects with previous intraocular surgery or laser treatment, penetrating eye injury, or corneal disorders preventing anterior chamber assessment. This study was conducted with the approval from the SingHealth Institutional Review Board, in accordance with the Declaration of Helsinki, with written informed consent obtained from all subjects before participation.

For this study we obtained consecutive, anterior segment scans of the right eye from each participant using the AS-OCT (Visante 3.0, Carl Zeiss Meditec, Dublin, CA) under standardized conditions of light (20 lux) by an operator who was masked to the results and other clinical examination findings.[17] Scans were centered on the pupil and taken along the horizontal axis (nasal-temporal angles at 0–180 degrees) using the standard anterior segment single-scan protocol to maximize visibility of anatomic location and repeatability.[18] The ZAAP software was then used to assess all AS-OCT images using an algorithm previously described,[7] where the only observer input was to determine the location of the 2 scleral spurs in each image (WC). The scleral spur was defined as the anatomic junction between the inner wall of the trabecular meshwork and the sclera.[19] The algorithm automatically derived the anterior segment and corneal parameters: ACD (from posterior corneal surface), central corneal thickness (CCT), ACC, PCC and PCAL.[5], [7]

### Statistical Methods

We used the mean with standard deviation (SD) for continuous anterior segment variables. Mean differences in measurements between groups were assessed using analysis of variance or independent samples t-test, after tests for normality where appropriate. Pearson's correlation coefficients (r) were calculated to describe the strength of the linear relationship between various anterior segment parameters, such as between PCAL, PCC and the various anterior segment parameters and systemic parameters. Estimated marginal means (EMM) of the anterior segment parameters, adjusted for age, gender and ethnicity, were computed and compared among ethnic groups using Bonferroni correction, presented with their standard error (SE) i.e. the general linear model was used to calculate the mean response for each factor, and adjusted for other variables in the model. Multiple linear regressions were used to assess the determinants and parameters' relationship with PCAL and PCC. Primary variables modeled included age, gender and ethnicity, and multivariate variables included age, gender, ethnicity, ACD, sphere, cylinder and intraocular pressure (IOP). Stepwise multiple linear regressions were used to determine the determinants of PCAL and PCC, using stepwise criteria of probability-of-F-to-enter ≤0.050 and probability-of-F-to-remove ≥0.100. All reported P values were compared to a significance level of 5%. All analyses were performed using SPSS version 20 (IBM Corporation, Armonk, NY, USA).

## Results

A total of 1118 eyes were included in our study, of which 1069 (96.0%) had identifiable scleral spurs. The mean age of our subjects was 56.9±9.5 years and 50.9% were male. The Chinese, Indian and Malay ethnic proportions were 29.9%, 46.4% and 23.7% respectively. Demographics of our study subjects, with their stratified anterior segment parameters (including PCAL, PCC) are described in **Table 1**. The mean age of the Chinese, Indian and Malay groups were 55.9±8.7, 57.5±9.5 and 56.8±10.3 years respectively (P = 0.046). There were no significant differences in gender proportions in each ethnic group (male, Chinese: 53.4%, Indian: 48.6%, Malay: 52.2%; P = 0.359). In summary, the overall mean (± SD) of the anterior segment parameters of our study cohort respectively were – PCAL 12.52±0.59 mm and PCC 6.51±0.39 mm. ACD, CCT, PCC and ACC showed a significant decreasing trend with increasing age (P<0.001), while males were noted to have a statistically significant higher PCAL, ACD, PCC and ACC (P<0.001).

**Table 1:** ZAAP measurements of Posterior Corneal Arc Length, Posterior Cornea Curvature and Corneal parameters, by Age, Gender and Ethnicity.

Characteristics	*n*	Corneal Parameters									
		PCAL (mm)		PCC (mm)		CCT (µm)		ACD (mm)		ACC (mm)	
		Mean	(SD)	Mean	(SD)	Mean	(SD)	Mean	(SD)	Mean	(SD)
All persons	1069	12.52	0.59	6.51	0.39	560.75	35.07	2.73	0.35	7.24	0.41
Age											
40–49	301	12.59	0.57	6.55	0.38	566.58	37.31	2.85	0.33	7.28	0.38
50–59	363	12.54	0.59	6.56	0.40	561.79	34.39	2.75	0.34	7.28	0.37
60–69	281	12.43	0.62	6.46	0.38	558.30	33.36	2.62	0.36	7.18	0.44
≥70	124	12.45	0.55	6.43	0.44	549.10	32.15	2.59	0.31	7.14	0.52
*P*-value for trend*		**0.009**		**<0.001**		**<0.001**		**<0.001**		**<0.001**	
Gender											
Male	544	12.58	0.59	6.55	0.38	559.22	34.70	2.79	0.35	7.28	0.40
Female	525	12.45	0.58	6.47	0.40	562.33	35.42	2.66	0.34	7.19	0.42
*P*-value*		**<0.001**		**0.001**		0.147		**<0.001**		**<0.001**	
Ethnicity											
Chinese	320	12.49	0.54	6.44	0.36	567.85	33.43	2.71	0.33	7.15	0.34
Indian	496	12.41	0.59	6.43	0.37	561.81	34.00	2.72	0.37	7.16	0.40
Malay	253	12.75	0.59	6.77	0.37	549.69	36.58	2.77	0.34	7.50	0.42
*P*-value*		**<0.001**		**<0.001**		**<0.001**		0.091		**<0.001**	

*based on analysis of variance or independent-samples t-test where appropriate.

ZAAP: zhongshan assessment programme; PCAL: posterior corneal arc length; ACD: anterior chamber depth; CCT: central corneal thickness; PCC: posterior corneal curvature; ACC: anterior corneal curvature;

SD: standard deviation.

We summarized the relationship of PCAL and PCC to the other measured corneal and anterior segment parameters between ethnic groups in **Table 2**. There was a positive correlation between PCAL and ACD (r = 0.546, P<0.001), across all three ethnic groups – Chinese (r = 0.508, P<0.001), Indian (r = 0.600, P<0.001) and Malay (r = 0.481, P<0.001). We also found weak correlations of other corneal parameters with PCAL, which included, PCC (r = 0.269, P<0.001), ACC (r = 0.136, P<0.001) and CCT (r = −0.070, P = 0.023). There was positive correlation between PCC and ACC (r = 0.814, P<0.001), across all three ethnic groups – Chinese (r = 0.774, P<0.001), Indian (r = 0.868, P<0.001) and Malay (r = 0.651, P<0.001). Weak correlation between PCC and CCT was found in Malay (r = 0.133, P = 0.035). Estimated marginal means (EMM) of the anterior segment parameters, adjusted for age, gender and ethnicity, were computed and compared among the ethnic groups – **Table 3** **.** We found that Malays have a relatively higher PCAL (EMM = 12.74mm, SE = 0.04 mm) than Chinese (EMM = 12.48 mm, SE = 0.03 mm, P<0.001) and Indians (EMM = 12.42 mm, SE = 0.03 mm, P<0.001).

**Table 2:** Pearson's correlations between ZAAP measurements of PCAL, PCC and corneal parameters, and by ethnicity.

PCAL & PCC	Corneal parameters			
	PCC (mm)	ACD (mm)	CCT (µm)	ACC (mm)
PCAL (*n* = 1069)				
Pearson's correlation	0.269	0.546	−0.070	0.136
*P*-value	**<0.001** *	**<0.001** *	**0.023** *	**<0.001** *
Ethnicity				
Chinese (*n* = 320)				
Pearson's correlation	0.175	0.508	−0.056	0.037
*P*-value	**0.002** *	**<0.001** *	0.315	0.513
Indian (*n* = 496)				
Pearson's correlation	0.258	0.600	−0.050	0.102
*P*-value	**<0.001** *	**<0.001** *	0.265	**0.023** *
Malay (*n* = 253)				
Pearson's correlation	0.150	0.481	0.009	0.018
*P*-value	**0.017** *	**<0.001** *	0.891	0.778
PCC (*n* = 1069)				
Pearson's correlation	-	0.053	−0.055	0.814
*P*-value	**-**	0.085	0.074	**<0.001** *
Ethnicity				
Chinese (*n* = 320)				
Pearson's correlation	-	0.071	−0.018	0.774
*P*-value	-	0.204	0.752	**<0.001** *
Indian (*n* = 496)				
Pearson's correlation	-	-0.011	−0.042	0.868
*P*-value	-	0.810	0.353	**<0.001** *
Malay (*n* = 253)				
Pearson's correlation	-	0.075	0.133	0.651
*P*-value	-	0.235	**0.035** *	**<0.001** *

ZAAP: zhongshan assessment programme; PCAL: posterior corneal arc length; PCC: posterior corneal curvature; ACD: anterior chamber depth; CCT: central corneal thickness; ACC: anterior corneal curvature.

*Significant P-value <0.05.

**Table 3:** Estimated marginal means (EMM) of ZAAP measurements of Posterior Corneal Arc Length, Posterior Cornea Curvature and Corneal parameters.

Ethnicity	Age-Gender-Ethnicity adjusted									
	PCAL (mm)		PCC (mm)		CCT (µm)		ACD (mm)		ACC (mm)	
	EMM	(SE)	EMM	(SE)	EMM	(SE)	EMM	(SE)	EMM	(SE)
Chinese	12.48	(0.03)	6.43	(0.02)	567.45	(1.91)	2.69	(0.02)	7.14	(0.02)
Indian	12.42	(0.03)	6.43	(0.02)	562.12	(1.53)	2.73	(0.01)	7.16	(0.02)
Malay	12.74	(0.04)	6.77	(0.02)	549.70	(2.15)	2.76	(0.02)	7.50	(0.02)
*P*-value*										
Chinese *vs.* Indian	0.499		1.000		0.091		0.338		1.000	
Chinese *vs.* Malay	**<0.001**		**<0.001**		**<0.001**		**0.026**		**<0.001**	
Indian *vs.* Malay	**<0.001**		**<0.001**		**<0.001**		0.494		**<0.001**	

*Bonferroni corrected.

ZAAP: zhongshan assessment programme; PCAL: posterior corneal arc length; ACD: anterior chamber depth; CCT: central corneal thickness; PCC: posterior corneal curvature;

ACC: anterior corneal curvature; SD: standard error.

We also performed a stepwise multiple linear regression and found that factors such as age, gender, ethnicity, ACD, PCC and IOP were important determinants of PCAL, with ACD being the most important determinant (partial R^2^  = 0.300; P<0.001) – **Table 4**. The stepwise multiple linear regression model was able to explain 37.6% variation of PCAL: PCAL  = 7.722+0.910*ACD +0.333*PCC +0.112 (if ethnicity was Malay) + 0.005*Age –0.076 (if ethnicity was Indian) – 0.010*IOP. A similar stepwise multiple linear regression was performed for PCC, and factors such as ACC, PCAL, CCT and cylinder were found to be important determinants of PCC, with ACC being the most important determinant (partial R^2^  = 0.683; P<0.001) – **Table 5**. The stepwise multiple linear regression model was able to explain 72.1% variation of PCC: PCC  = −0.699+0.752*ACC +0.123*PCAL +0.0005*CCT +0.021*Cylinder.

**Table 4:** Stepwise multiple linear regression analysis on ZAAP measurements of Posterior Corneal Arc Length and parameters*.

No. of variables in model	Variable	Regression coefficient	(95% CI)		Adjusted R^2^	Partial R^2^	P-value
1	ACD (mm)	0.91	(0.83,	0.99)	0.291	0.300	**<0.001**
2	PCC (mm)	0.33	(0.25,	0.41)	0.355	0.060	**<0.001**
3	Ethnicity - Malay (Chinese as reference)	0.11	(0.03,	0.20)	0.365	0.007	**0.008**
4	Age (years)	0.01	(0.002,	0.01)	0.371	0.011	**0.001**
5	Ethnicity - Indian (Chinese as reference)	−0.08	(−0.14,	−0.01)	0.374	0.005	**0.026**
6	IOP (mmHg)	−0.01	(−0.02,	−0.001)	0.376	0.004	**0.036**

*using stepwise criteria of probability-of-F-to-enter ≤0.050 and probability-of-F-to-remove ≥0.100.

ZAAP: zhongshan assessment programme; CI: confidence interval; ACD: anterior chamber depth; PCC: posterior corneal curvature; IOP: intraocular pressure.

**Table 5:** Stepwise multiple linear regression analysis on ZAAP measurements of Posterior Corneal Curvature and parameters*.

No. of variables in model	Variable	Regression coefficient	(95% CI)		Adjusted R^2^	Partial R^2^	P-value
1	ACC (mm)	0.75	(0.72,	0.78)	0.684	0.683	**<0.001**
2	PCAL (mm)	0.12	(0.10,	0.14)	0.719	0.110	**<0.001**
3	CCT (µm)	0.0005	(0.0001,	0.001)	0.720	0.006	**0.012**
4	Cylinder (D)	0.02	(0.002,	0.04)	0.721	0.004	**0.031**

*using stepwise criteria of probability-of-F-to-enter ≤0.050 and probability-of-F-to-remove ≥0.100.

ZAAP: zhongshan assessment programme; CI: confidence interval; ACC: anterior corneal curvature; PCAL: posterior corneal arc length; CCT: central corneal thickness; D: dioptre.

## Discussion

Our current study found significant differences in PCAL between the three major Asian ethnic groups, with Malays having higher PCAL EMM than Chinese and Indians (P<0.001), still significant even after adjusting for factors such as age, gender and other anterior segment parameters. These differences may have impact on our clinical practice, such as intra-operative graft sizing for endothelial keratoplasty, where currently measurements are based on visual estimation of the horizontal white-to-white diameter that does not take into account the cornea curvature.[20] Using the AS-OCT-ZAAP to derive the PCAL in different ethnicities may have clinical impact on the choice of the graft size where Malays who have a relatively higher PCAL (EMM = 12.74 mm) than Chinese (EMM = 12.48 mm, P<0.001) and Indians (EMM = 12.42 mm, P<0.001) could essentially have larger endothelial keratoplasty grafts (up to 30% estimated from previous studies).[20] Other clinic implications of posterior corneal measurements include: the impact of the posterior cornea curvature on the total astigmatism,[21] especially for patients for toric intraocular lens implantation;[22] or even measurements of other parameters such as the Descemet membrane length (study in progress). Moreover, we also found that the most important determinants of PCAL were ACD and PCC, after adjusting for age, gender, ethnicity and anthropometric data. However, the weak-moderate relationship between PCAL and these parameters suggests that PCAL is an independent parameter, which cannot be entirely predicted from other anterior segment measurements (adjusted R^2^  = 0.376).

We also analyzed the determinants of PCC in this study, and derived a formula that explains 72.1% of the variation of PCC. We found that ACC, PCAL, and CCT were the most significant determinants of PCC. Moreover, we observed significant inter-ethnic differences in PCC (Malays had higher ACC and PCC compared to Chinese, P<0.001; and Indians, P<0.001). This suggests that in Asian eyes, ethnic based adjustments to calculations for IOL power may be needed, as the posterior corneal power is increasingly being recognized as an important factor for accurate refractive measurements.[23], [24] The observed significant differences in PCAL and PCC amongst factors such as age, gender and ethnicity, suggest that these require some consideration when planning anterior segment surgery i.e. such as a young Malay male would likely have significantly different anterior segment dimensions and posterior cornea surface from an elderly Indian female when planning for anterior segment surgery.

We also studied inter-ethnic differences in anterior segment parameters specifically amongst Asians. We found that Chinese and Indians had thicker EMM CCT than Malays (P<0.001) using ZAAP analysis of AS-OCT images. This is not consistent with previous studies, which found that Indians had thinner corneas than Caucasians or Chinese but the difference maybe due to differences in study methodology. [25], [26]. It may also be due to the fact that CCT may be affected by genetic as well as environmental factors.[27], [28], [29] Clinicians should keep this in mind when planning for corneal refractive surgery especially in ethnic Chinese and Indians,[30] where pre-operative CCT measurements are crucial considerations for corneal/refractive procedures.[31] Our central ACD measurements using ZAAP EMM were lower in our Chinese subjects compared to Malays (P = 0.026). Shallow ACD in Chinese subjects has been previously documented in comparison to a Caucasian cohort (28). We also found that the mean radius of ACC in our study cohort was 7.36 ± 0.41 mm, with a greater ACC in males compared to female, similar to another predominantly Caucasian population study cohort.[32]

While comparisons between Asian and Caucasian eyes have shown that Asian eyes have smaller anterior segment parameters,[33], [34] this is the first population-based study that provides direct inter-ethnic AS-OCT corneal parameter comparisons, specifically amongst Asians.[34], [35] The strengths of our study include a large sample size with standardized ocular assessment by ophthalmologists, using an objective measurement of anterior segment parameters using the ZAAP analysis. The clinical impact of anterior segment assessment is increasingly being recognized, for planning of surgical procedures,[9] pre-operative evaluation for corneal refractive procedures,[31] phakic IOL implantations,[36] biometric IOL calculations,[37] and for deciding on donor graft size for endothelial keratoplasties.[7] We recognize the limitations of ZAAP and AS-OCT imaging due to the inability to detect the scleral spur in suboptimal images, or where the sclera formed a smooth continuous line - we only used horizontal nasal–temporal AS-OCT scans as these have been shown to be the most consistent for the ZAAP software to analyze. In this study, we found that the anterior segment parameters studied only explained 37.6% of the variance of PCAL – which needs further research to look for known and unknown factors which may affect PCAL measurements. We also acknowledge that although we compared our posterior corneal measurements among the three Asian groups, data from three cross-sectional population-based studies were used with slightly different study periods and sample sizes – however, it would be difficult to perform the ‘ideal’ prospective cohort study involving all three ethnic groups with coinstantaneous recruitment as well as equal and large sample sizes. Our study observations need to be confirmed in larger, direct comparative studies between the different ethnicities or ethnic-subgroups

In summary, in this study we found that PCAL, which gives an estimation of the internal corneal diameter, is relatively independent measurement of the posterior cornea that cannot be determined by other anterior segment parameters. The posterior corneal power however, was significantly affected by the ACC, PCAL and corneal thickness in our study cohort. The significant differences in PCAL and PCC between age, gender and Asian ethnic groups suggests that there is a need to consider this factor when planning for anterior segment surgeries like donor graft size selection in endothelial keratoplasties.
